# Editorial: Current Aspects in Chemopreventive Strategies, Volume II

**DOI:** 10.3389/fphar.2022.961334

**Published:** 2022-07-15

**Authors:** Hardeep Singh Tuli, Mükerrem Betül Yerer, Katrin Sak

**Affiliations:** ^1^ Department of Biotechnology, Maharishi Markandeshwar (Deemed to be University), Ambala, India; ^2^ Department of Pharmacology, Faculty of Pharmacy, Erciyes University, Kayseri, Turkey; ^3^ NGO Praeventio, Tartu, Estonia

**Keywords:** anti-cancer agents, apoptosis induction, anti-angiogenesis, anti-metastasis, anti-inflammation

Despite extensive studies, cancer remains one of the most dreadful diagnoses and biggest challenges for human health all over the world, representing a leading cause of death in the industrialized countries. Various chemotherapeutic drugs, such as doxorubicin, tamoxifen and paclitaxel, have been used for the treatment of tumors for more than half a century; however, there are still no curative options currently available in clinical settings and the severe adverse effects of these drugs threaten the well-being of the patients seriously. Current evidence suggests that further knowledge is urgently needed to clarify the unknown properties and molecular mechanisms of action of various chemopreventive molecules. Such substances refer to the agents which are used for reducing the risk of carcinogenesis, or delaying development or recurrences of malignant disorders.

Several small molecules either of biological origin or from synthetic chemistry, such as cordycepin, mitomycin C, doxorubicin and methotrexate, have demonstrated great efficacy towards a variety of cancers. These agents are found to suppress the proliferation of cancer cells by various mechanisms such as apoptotic cell death, cancer cell cycle arrest, inhibition of angiogenesis and metastasis, induction of ROS generation etc. Few *in silico* tools, such as docking and QSAR (Quantitative structure-activity relationship) techniques, can be used to retrieve more comprehensive information about the targets and the action mechanisms of such molecules. However, there has been a vigorous need to explore the acute as well as the chronic toxicological effects of such chemopreventive molecules for further clinical implementation against diseases.

This Research Topic aims to highlight the ongoing advancement in chemopreventive and therapeutical approaches, as well as the promising role of the above-mentioned agents in the context of cancer prevention and therapy. In particular, it encompasses the research of Singh et al. about potential therapeutic significance of 4-(methylthio) butyl isothiocyanate (4-MTBITC) on modulation of glycolytic enzymes and hypoxia pathway in female rats. As increased glycolysis is known to be an indicator of malignancies (Zhong et al., 1999), inhibition of glycolytic enzymes by 4-MTBITC might be important in delaying the tumor progression. A review carried out by Kumar et al. describes chemopreventive and anticancer potential of kurarinone, presenting also isolation, bioavailability, metabolism and toxicity of this natural flavanone in different experimental models. Another research conducted by Singh et al. by using *Berberis aquifolium root* mother tincture (BAMT) shows multi-pronged therapeutic potential of this extract against HPV infection and cervical cancer. Infection with high-risk HPV subtypes is generally accepted as a risk factor for cervical carcinogenesis (Bharti et al., 2018), highlighting the potential role of anti-HPV action of BAMT in cervical cancer prevention. Abdalla et al. demonstrate safranal as a potent chemopreventive agent against hepatocellular carcinoma. Similarly in another study, Abdalla et al. explore antiangiogenic potential of safranal and propose its possible underlying mechanism in hepatocellular carcinoma (HCC). In a further article, Baba et al. review the dual role of TGF-β under different cellular conditions and its crosstalk with other signaling pathways in modulating cell fate. As a pleiotropic cytokine, TGF-β can act both as a tumor suppressor as well as tumor promoter depending on the context and stage of tumor progression (Yang and Moses, 2008). Fined-tuned modulation of this factor can therefore retard carcinogenesis process. Hakroush et al. describe a case report on Ado-trastuzumab emtansine uses in a breast cancer patient, analyzing nephrological aspects. As this therapy is widely applied in present-day oncological settings, clinicians should be aware of severe nephrological complications associated with administration of this antibody-drug conjugate. Mahapatra et al. suggest phenethylisothiocyanate as a potential chemosensitizing agent in light of acquired cisplatin resistance in cervical cancer and established its candidature for Phase I clinical trial. Shankar et al. and Farabegoli et al. describe a strategic approach in cancer chemoprevention using structurally diverse phytochemicals including curcumin, resveratrol, gingerol, epigallocatechin 3-gallate (EGCG), quercetin and grape seed procyanidins, among several other bioactive natural compounds, in the models of different cancer types. Hu et al. describe the contribution of lncRNAs and miRNAs in triple-negative breast cancer pathogenesis, proliferation, migration or malignancy. Finally, Rah et al. review the role of JAK/STAT pathway to improve the existing cancer therapies. Modulation of this signaling cascade might be important also in cancer prevention. We hope that you enjoy the reading of this article collection, composed in a topic that will probably be even more important in the coming years.
